# An Augmented Reality Geo-Registration Method for Ground Target Localization from a Low-Cost UAV Platform

**DOI:** 10.3390/s18113739

**Published:** 2018-11-02

**Authors:** Xiang Ren, Min Sun, Cheng Jiang, Lei Liu, Wei Huang

**Affiliations:** 1 Institute of Remote Sensing and GIS, Peking University, Beijing 100871, China; xelmirage@pku.edu.cn (X.R.); liuleiargis@pku.edu.cn (L.L.); 2 Netease Network Co. Ltd., Hangzhou 310052, China; jiangcheng0823@163.com; 3 The 61206 Troop, PLA, Beijing 100042, China; 4 GIScience Research Group, Institute of Geography, Heidelberg University, Im Neuenheimer Feld 368, 69120 Heidelberg, Germany; huangweibuct@gmail.com

**Keywords:** augmented reality, geo-registration, UAV, target localization, RTK GPS

## Abstract

This paper presents an augmented reality-based method for geo-registering videos from low-cost multi-rotor Unmanned Aerial Vehicles (UAVs). The goal of the proposed method is to conduct an accurate geo-registration and target localization on a UAV video stream. The geo-registration of video stream requires accurate attitude data. However, the Inertial Measurement Unit (IMU) sensors on most low-cost UAVs are not capable of being directly used for geo-registering the video. The magnetic compasses on UAVs are more vulnerable to the interferences in the working environment than the accelerometers. Thus the camera yaw error is the main sources of the registration error. In this research, to enhance the low accuracy attitude data from the onboard IMU, an extended Kalman Filter (EKF) model is used to merge Real Time Kinematic Global Positioning System (RTK GPS) data with the IMU data. In the merge process, the high accuracy RTK GPS data can be used to promote the accuracy and stability of the 3-axis body attitude data. A method of target localization based on the geo-registration model is proposed to determine the coordinates of the ground targets in the video. The proposed method uses a modified extended Kalman Filter to combine the data from RTK GPS and the IMU to improve the accuracy of the geo-registration and the localization result of the ground targets. The localization results are compared to the reference point coordinates from satellite image. The comparison indicates that the proposed method can provide practical geo-registration and target localization results.

## 1. Introduction

UAV-based target monitoring plays an integral part in multiple areas such as traffic management [1,2,3], forest-fire control [4,5], border and port patrolling [6], wild animal tracking [7] and emergency management [8]. Conventional monitoring methods include fixed station monitoring, satellite monitoring and human-crewed aircraft monitoring [1]. Pinned stations are easy to set up and able to acquire various types of data, but the field of view is limited. Satellites can monitor vast areas, but their revisit time is too long for time-sensitive monitoring missions due to the orbit limitations. A single satellite needs typically tens of hours to revisit a particular target area, and thus tracing a moving target is nearly impossible. Instead, human-crewed aircraft are responsive in monitoring tasks, and the onboard sensors can satisfy the spatial and temporal resolution requirements. The main drawback of human-crewed aircrafts is the high cost, and, considering the safety of the pilots, human-crewed aircrafts cannot operate in hazardous environments.

As an emerging platform, UAVs have significant advantages in monitoring tasks [9,10]. They have higher mobility than pinned stations, which allows a broader field of view, lower cost and risk than human-crewed aircrafts, with higher time and spatial resolution. The fast-growing market of low-cost electronic multi-rotor UAVs has dramatically reduced the difficulty of using UAV platforms. Thus using such low-cost UAV platforms to obtain information of target area is being popular for researchers. A major problem needs to be solved in related research areas is how to assist the ground operators in obtaining information and establishing the situation awareness of the working zone when a UAV is airborne and monitoring the ground objects.

Augmented Reality (AR) is a promising technology to solve this problem. AR can overlay the known information such as vector map, raster map and other attributes of the ground objects in the corresponding locations on the screen by registering virtual 3D objects to the video recording the physical world. The geographic coordinates and the 3-axis attitude of the onboard camera are required to augment the known information on the video from UAVs. The accurate measurement of the camera attitude needs navigation-level IMU, which is not feasible in cost and weight to be used on low-cost electronic multi-rotor UAVs. It is difficult to directly apply navigation-level IMU on low-cost UAVs. In the meantime, high accuracy RTK GPS receivers are becoming popular as the weight and cost decrease. Thus utilizing the high accuracy RTK GPS data to augment the original attitude data is a reasonable way of acquiring accurate attitude data for augmented reality geo-registration.

In accordance with the drawbacks of existing researches, this research focuses on using high precision RTK GPS data to reduce the attitude error from the low-cost onboard IMU for a better AR geo-registration and ground target localization.

## 2. Related Research

As a target-monitoring platform, UAV systems have apparent advantages along with considerable challenges. When performing remote monitoring tasks, ground operators can hardly obtain sufficient information for identifying and monitoring targets [11,12]. Calhoun [13] used augmented reality to overlay models of the landing zone, runway, and buildings in the real-time video stream for pilots to emphasize critical spatial information and assist monitoring the target area. However, the focus of this research was to analyze available types of spatial information, and possible manners of presenting the information, the precision of registration was not well discussed. Crowley [14] pointed out that it was difficult for rescue personnel to identify important or obstructed buildings, they need to compare the video stream from UAV to paper maps or electronic maps, which will waste plenty of time and cause certain potential errors. To solve this problem, they overlaid the images from UAV on Google Maps and marked Points of Interest (POIs). Limited by the process capability of the mobile platforms, the system could only process static images. Drury [15] used within-subject design to experiment on the effectiveness of UAV remote monitoring systems with augmented reality technology. During the experiment, the participants watched the original video stream from UAVs and augmented video stream that with and without the terrain information separately and then completed a rescuing task. The experiment result indicated that when the video was augmented by the terrain information, the participants were able to identify more targets and the positioning of rescue targets was more accurate.

Therefore, augmented reality is proven to have the ability to solve the problem of a lack of information during UAV target monitoring and promote the situation awareness of ground operators [16,17]. Augmented reality overlays known information of the ground objects on the video in the correspondent positions through conducting geo-registration. Geo-registration (or geo-referencing) defines that overlaying virtual scenes on an actual video stream using the pose data of the camera, which is the key factor of outdoor augmented reality. Eugster [18,19,20] described the method of geo-registering video stream from UAV platforms and proposed a two-phase geo-referencing model, including direct geo-referencing and integrated geo-referencing. Direct geo-referencing means directly using the GPS/IMU data provided by a UAV as the exterior orientation elements. Integrated geo-referencing uses additional observations derived from images to known control points to estimate the exterior orientation elements of the camera. The experiment results indicated that when the flying height of UAVs is 50 m, the direct geo-referencing error was around 3 m, and the error of integrated geo-referencing was able to achieve 0.6 m. Despite integrated geo-referencing achieved sub-meter accuracy, the complicated operations such as edge detection, feature point extraction and Hough transformation limited the application on small UAV platforms. Meanwhile, direct geo-registration is widely used for its low computational load and well real-time ability. Ruano [21] proposed an augmented tool for situational awareness of the ground operators. However, this research didn’t validate the registration accuracy. Stilla [22] used direct geo-referencing to make the thermal texture of buildings. When the flying height was 400 m, the accuracy was 4 m, which is sufficient for LOD2 (Level of Detail) city modeling. However, the rest platform of this research was a human-crewed helicopter and the high accuracy IMU was not suitable to be used on low-cost UAVs. Eugster [18] used extended Kalman Filter to merge GPS, IMU, barometer and other data to obtain a better estimation of the UAV and realize a better geo-registration result. Nevertheless, the IMU used in this research was also too cumbersome and expensive for electronic multi-rotor UAVs.

Augmented reality provides the ability to identify ground targets for UAV monitoring systems, therefore the target localization technology enables extended applications of target monitoring technology. Target localization means estimating the actual geographical location of targets in real-time using various types of sensors onboard UAVs. In principle, there are two types of methods for target localization: the first is based on image matching and the second uses the GPS/IMU data provided by UAVs. The image matching based method corrects the geometry distortion and radiation distorting of the images, and if necessary, the images will be filtered. After these processes, an image is matched with an existing standard map and locates the targets [23]. The high computational cost and the needs of pre-obtained orthoimages of a target area limit the application of this method in target monitoring, although this method is remarkably reliable. The GPS/IMU data-based method determines the image space coordinates of the target using the image point coordinates and calculates the actual geographic coordinates of the targets from the relations between the image space coordinate system, the UAV coordinate system, and the geodetic coordinate system. This type of method can be further divided into triangulation based approaches [24,25], laser ranging based approaches and DEM(Digital Elevation Model) based approaches [26]. Barber [25] presented a triangulation based method of ground targets localization from a fixed-wing miniature air vehicle. In spite of that the localization error can be reduced down to less than 5 m, the method needs a circular trajectory around the target, which makes this method impractical in the most circumstances. Ponda [27] described the GPS/IMU data-based method in detail and pointed out that the low-cost and low-accuracy GPS/IMU was the main source of error. Thus, despite that this type of method is simple to implicate and has low computational cost, it is not accurate enough. To solve this problem, Ponda et al. increased the target localization accuracy through optimized UAV trajectory and adjusting the trajectory in real-time. However, this also increased the workload of the ground operators. Our previous work [28] introduced user interaction to correct the registration error during the augmentation process, the method provided satisfying results along with heavy user burden which made it not feasible to be applied on UAV video streams. Therefore, either a computational expensive feature matching process or navigation-level IMU is needed in existing researches to acquire high accuracy attitude data for the geo-registration process, which is not feasible to be applied on low-cost multi-rotor UAVs.

In order to overcome the drawbacks of existing augmented reality geo-registration methods and get a reliable and high accuracy registration result for videos from low-cost UAVs, a new method is proposed in this paper. The proposed method provides a whole solution of augmenting video streams from low-cost UAV platforms and ground target localization. The core idea of the proposed method is using high accuracy RTK GPS data to improve the attitude data, which can make the registration process and the target localization process more accurate and stable, and at the same time avoid high computational cost image-matching process. 

## 3. Methodology

### 3.1. The Coordinate Conversion in Augmented Reality Geo-Registration

3D geo-registration is the process to project the objects in a virtual scene to the corresponding environment of the real world. Mostly, it implies a conversion between the world coordinate system and the screen coordinate system. There are four coordinate systems will be involved in the process of geo-registering the UAV video stream:

- The world coordinate system W: The geographical coordinate system. The GPS data and the ground vector map is in this coordinate system. In this research, W denotes the WGS-84 coordinate system.
- The camera coordinate system C: The origin of the coordinate system is the optical center of the lens. This coordinate system is used to relocate the objects in the world coordinate system from the perspective of observation.
- The projection surface coordinate system P: This coordinate system is a two-dimensional coordinate system, which is used to define the projection for the points of objects.
- The screen coordinate system S: The origin of this coordinate system is the upper left point of the screen.

**Figure 1:**
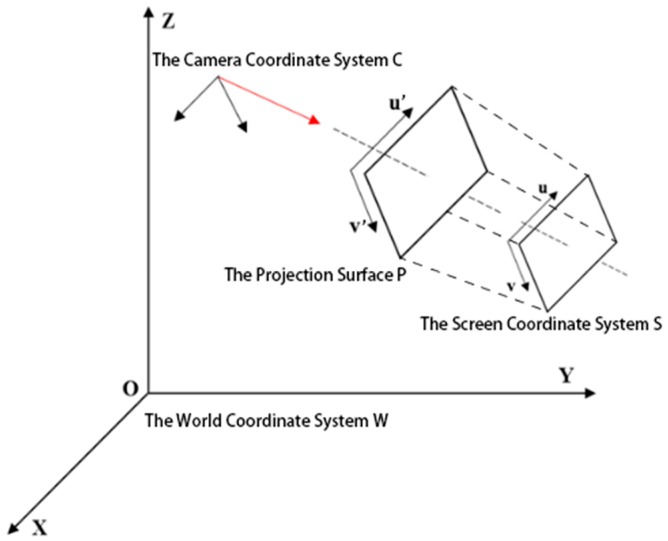
The relative position relations of the coordinates. The O-XYZ coordinate system is the world coordinate system; C is the camera coordinate system; P is the projection surface coordinate system; and S is the screen coordinate system.

Figure 1 shows the relative positional relations between the coordinates.

**Figure 2:**
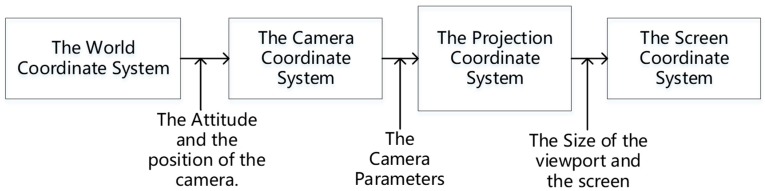
The conversion process between the world coordinate system and the screen coordinate system.

As shown in Figure 2, the coordinates in the real world coordinate system W will be converted successively to C, P, and S.

The location of ground objects in the world coordinate system is determined. During the flight, the coordinates of the UAV and the poses of the camera can be obtained in real time. To simplify the model, the latitude and longitude coordinates are projected by Gauss projection [29]:

(1) $$\{\begin{array}{c} x=X+\frac{Nl^{2}}{2\rho^{2}}\sin B\cos B+\frac{N\left(5-t^{2}+9\eta^{2}+4\eta^{4}\right)l^{4}}{24\rho^{4}}\sin B\cos^{3}B+\frac{N\left(61-58t^{2}+t^{4}\right)l^{6}}{720\rho^{6}}\sin B\cos^{5}B\\ y=\frac{Nl}{\rho }\cos B+\frac{N\left(1-t^{2}+\eta^{2}\right)l^{3}}{6\rho^{3}}\cos^{3}B+\frac{N\left(5-18t^{2}+t^{4}+14\eta^{2}-58\eta^{2}t^{2}\right)l^{5}}{120\rho^{5}}\cos^{5}B \end{array},$$

where B is the latitude; l is the difference between the longitude L and the central meridian $L_{0}$, $l=L-L_{0}$; X is the meridian arc length from the equator; N is the curvature radius of the prime vertical circle; t=tanB; $\eta =e^{\prime }\cos B$, $e^{\prime }$ is the second eccentricity of the standard spheroid; ρ=180/π·60·60.

Then the translation matrix from W to C is:

$$A=\left[\begin{array}{cc} \begin{array}{ccc} 1 & 0 & 0\\ 0 & 1 & 0\\ 0 & 0 & 1 \end{array} & \begin{array}{c} -x\\ -y\\ -h \end{array}\\ \begin{array}{ccc} 0 & 0 & 0 \end{array} & 1 \end{array}\right]$$

h is the flying height of the UAV. If we use γ, α and β to represent the roll, pitch, and yaw of the camera, the rotate matrixes of the three axes can be written as:

$$M_{1}=\left[\begin{array}{cccc} \cos\left(-\gamma \right) & 0 & -\sin\left(-\gamma \right) & 0\\ 0 & 1 & 0 & 0\\ \sin\left(-\gamma \right) & 0 & \cos\left(-\gamma \right) & 0\\ 0 & 0 & 0 & 1 \end{array}\right]=\left[\begin{array}{cccc} \cos\gamma & 0 & \sin\gamma & 0\\ 0 & 1 & 0 & 0\\ -\sin\gamma & 0 & \cos\gamma & 0\\ 0 & 0 & 0 & 1 \end{array}\right]$$

$$\;M_{2}=\left[\begin{array}{cccc} 1 & 0 & 0 & 0\\ 0 & \cos\left(-\alpha \right) & \sin\left(-\alpha \right) & 0\\ 0 & -\sin\left(-\alpha \right) & \cos\left(-\alpha \right) & 0\\ 0 & 0 & 0 & 1 \end{array}\right]=\left[\begin{array}{cccc} 1 & 0 & 0 & 0\\ 0 & \cos\alpha & -\sin\alpha & 0\\ 0 & \sin\alpha & \cos\alpha & 0\\ 0 & 0 & 0 & 1 \end{array}\right]\;$$

$$\;M_{3}=\left[\begin{array}{cccc} \cos\left(-\beta \right) & \sin\left(-\beta \right) & 0 & 0\\ -\sin\left(-\beta \right) & \cos\left(-\beta \right) & 0 & 0\\ 0 & 0 & 1 & 0\\ 0 & 0 & 0 & 1 \end{array}\right]=\left[\begin{array}{cccc} \cos\beta & -\sin\beta & 0 & 0\\ \sin\beta & \cos\beta & 0 & 0\\ 0 & 0 & 1 & 0\\ 0 & 0 & 0 & 1 \end{array}\right]\;$$

Thus, the conversion matrix from W to C is:

$$M_{W-C}=M_{3}M_{2}M_{1}A$$

The conversion from C to P is a transformation from a three-dimensional space to a two-dimensional space, which is also named as perspective projection. The viewing frustum is determined by the FOV (Field Of View), the aspect ratio of the projection plane, the near plane and the far plane. Only the objects in the viewing frustum are observable.

The projection matrix from C to P is:

$$M_{C-P}=\left[\begin{array}{cccc} \frac{cot\left(\frac{fov}{2}\right)}{aspect} & 0 & 0 & 0\\ 0 & cot\left(\frac{fov}{2}\right) & 0 & 0\\ 0 & 0 & -\frac{far+near}{far-near} & -\frac{2far\cdot near}{far-near}\\ 0 & 0 & -1 & 0 \end{array}\right]$$

where fov is the vertical field of view of the camera, near is the distance between the camera optical center and the near plane, far is the distance between the camera optical center and the far plane, and aspect is the aspect ratio of the projection surface.

The project surface P and the screen S are on the same surface but using different origins and units. In the projection surface coordinate system P, the origin is the cross point of the main axis and the projection surface, and the unit is the physical unit. In the screen coordinate system, the origin is the upper left point, and the unit is pixel.

In the screen coordinate system, when the center of the projection surface is $\left(u_{0},v_{0}\right)$, the physical size of one pixel is ($d_{u},\;d_{v}$), a point in the projection surface $\left(u^{\prime },\;v^{\prime }\right)$ and its coordinate on the screen coordinate system (u, v) satisfies:

$$\{\begin{array}{c} u=\frac{u^{\prime }}{d_{u}}+u_{0}\\ v=\frac{v^{\prime }}{d_{v}}+v_{0} \end{array}$$

The conversion matrix from P to S is:

$$M_{P-S}=\left[\begin{array}{cccc} \frac{1}{d_{u}} & 0 & 0 & u_{0}\\ 0 & \frac{1}{d_{v}} & 0 & v_{0}\\ 0 & 0 & 0 & 0 \end{array}\right]$$

After all the conversions between the four coordinate systems, a point in the real world coordinate system can be converted to the screen coordinate system. If the coordinate of the point in W is (X, Y, Z), its coordinate on the screen will be:

(2) $$\left[\begin{array}{c} u\\ v\\ 1 \end{array}\right]=M_{P-S}M_{C-P}M_{W-C}\left[\begin{array}{c} X\\ Y\\ Z\\ 1 \end{array}\right],$$

### 3.2. The Acquisition of High Precision GPS/IMU Data

The poor GPS/IMU data is the primary error source of the camera exterior orientation elements. Furthermore, the error of camera exterior orientation elements might affect the accuracy of the geo-registration and target localization process. Our research tries to increase the accuracy using both hardware and software manners. The hardware manner includes using high accuracy RTK GPS and IMU and the software manner includes error source analysis and the filtering the position and attitude data.

#### 3.2.1. High Accuracy RTK GPS

A set of high accuracy RTK GPS receivers is on board the UAV. The ground station of the RTK GPS receivers determines the its own precise location by continuously observing for a while. During the flight, the correction is calculated by the RTK ground station and transmitted to the UAV through the data link. The mobile station on board the UAV receives the correction. Meanwhile, it also receives the GPS signal from the satellites at the same time, which can make the localization error <0.1 m [30]. The ground station receives the RTK GPS data at the rate of 10 Hz, This rate is lower than common IMU and video. In the filter process the algorithm can combine the RTK GPS data and the IMU data, in the interval between two RTK GPS data frames, the RTK GPS data is extrapolated to keep up with the high frequency IMU and video data.

#### 3.2.2. Filter Process of the Data 

**Figure 3:**
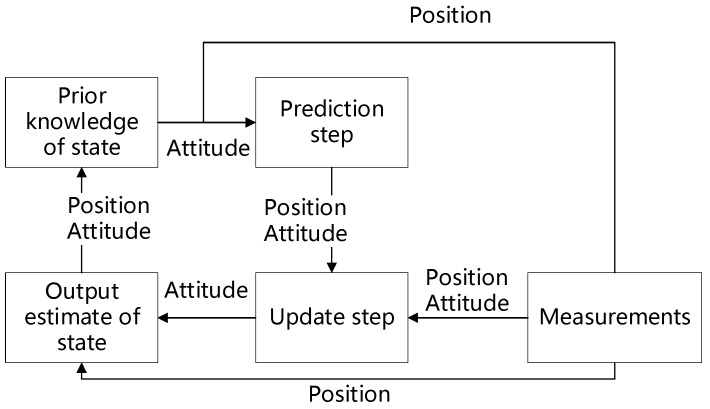
Diagram of the EKF algorithm applied in this research. The position measurement from the RTK GPS is directly used as the output of the filter process and the input of the next prediction step.

Since the UAV has high precision RTK GPS receivers, it is feasible to use the RTK GPS data to augment the altitude data. The Kalman Filter is proved to be a reliable way to merge data from multiple sensors to improve the data stability. In the Kalman Filter process, the status of the system is predicted using the previous observation, and the prediction is compared to the current measurement. The classic Kalman filter is only capable of describing linear systems. The extended Kalman Filter (EKF) linearizes a nonlinear system at the working position by a series of expansion, which allows the application of the Kalman filter on a nonlinear system. In this research, we use the EKF from González [31,32] to combine the data from RTK GPS and the IMU of the UAV to improve the accuracy of the geo-registration and the localization result of the ground targets. González [31,32] presented an approach to loosely couple a low-cost GPS receiver and a strapdown internal navigation system. The close-loop correction equations of the position are:

$${\hat{L}}_{b}\left(+\right)={\hat{L}}_{b}\left(-\right)+\delta {\hat{L}}_{b}$$

$$\;{\hat{\lambda }}_{b}\left(+\right)={\hat{\lambda }}_{b}\left(-\right)+\delta {\hat{\lambda }}_{b}\;$$

where ${\hat{L}}_{b}$ is the latitude, ${\hat{\lambda }}_{b}$ is the longitude, ${\hat{h}}_{b}$ is the height, $\delta {\hat{L}}_{b}$ is the latitude error, $\delta {\hat{\lambda }}_{b}$ is the longitude error, and $\delta {\hat{h}}_{b}$ is the height error. The (−) and (+) represents before and after correction. In this research, the application of RTK GPS provides high accuracy GPS coordinates. The original algorithm from [31,32] is designed for both low-cost INS and low cost GPS receiver, which means both the attitude and the position of the UAV have to be corrected by the filter. Since the RTK GPS has an extremely high precision, the GPS data is not changed by the algorithm and the original GPS data is used to predict the current status of the UAV and correct the status prediction in every iteration. Thus, in the EKF fusion process, only the attitude data of the UAV is modified, the position correction equations are modified to:

$${\hat{L}}_{b}\left(+\right)={\tilde{L}}_{g}\;$$

$$\;{\hat{\lambda }}_{b}\left(+\right)={\tilde{\lambda }}_{g}\;$$

where ${\tilde{L}}_{g}$ is the measurement of latitude from RTK GPS, ${\tilde{\lambda }}_{g}$ is the measurement of longitude from RTK GPS, and ${\tilde{h}}_{g}$ is the measurement of height from RTK GPS. This means the position measurements are directly used as the actual status of the system and output of the filter process. The whole process is illustrated in Figure 3.

Because the camera is mounted on the UAV using a brushless gimbal, the attitude of the camera cannot be determined directly by the attitude of the UAV body. The roll and the pitch angle of the camera are measures mainly using the gravity acceleration which is steady in normal conditions, the yaw angle of the camera is mainly measured by the magnetic compass, which is easy to be interference by the environment. Normally, the gimbal onboard the UAV keeps the same heading with the UAV body, the yaw angle of the UAV can be regarded as the yaw of the camera. Thus, after augmenting the roll, pitch and yaw angle of the UAV using the RTK GPS data, the yaw angle of the UAV can be used to replace the original yaw data of the camera and to improve the accuracy of the attitude data of the camera and the accuracy of the geo-registration.

### 3.3. The Target Localization Algorithm

The process of geo-registration uses pre-calibrated inner orientation parameters and exterior orientation elements obtained in real-time to adjust the project matrix and observing matrix, and then the markers in the virtual scene and objects in the real space can overlay in the same position, which can be divided into direct geo-referencing and integrated geo-referencing. Limited by the heavy computational load, integrated geo-referencing is not capable of real-time applications. Thus, we use direct geo-referencing. The camera is mounted on the UAV and has a certain downward sloping angle. The high precision GPS/IMU data is obtained by the dual antenna RTK GPS system and the onboard IMU. The data is transferred to the ground station to calculate the exterior orientation elements of the camera, which is needed by the geo-registration of the video stream.

**Figure 4:**
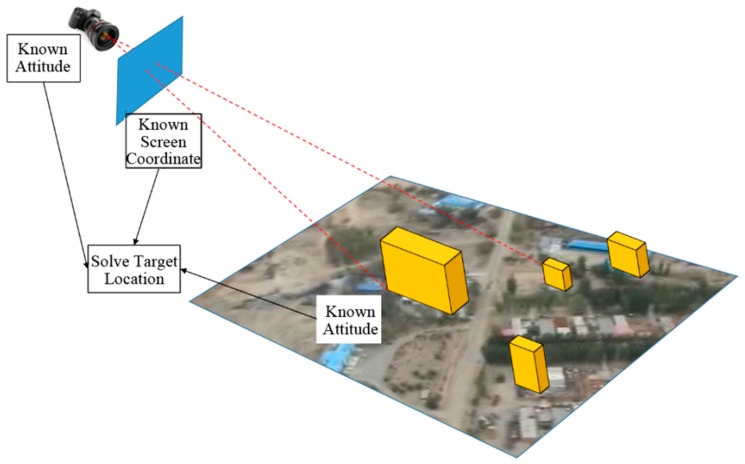
Target localization principle based on the GPS data and the attitude data of the UAV.

As mentioned in Section 2, according to the difference of supplementary data, the target localization methods can be divided into triangulation-based approaches [24], laser ranging-based approaches and DEM-based approaches [26]. Since the laser ranging method will significantly add up the weight of the UAV, we use the triangulation-based approach and the DEM-based approach. Figure 4 shows the target localization process of these two approaches.

If the exterior and interior orientation elements are known, the projection relationship between the real scene and the video stream can be calculated. According to the collinear principle, selecting a point from the video, make a ray connecting the optical center and the video point, and the half line will intersect with the land surface at the real location of the target. This process needs additional information to avoid the one-to-many problem such as the altitude of the target or the DEM data of the target zone.

However, the DEM data of the target zone is not always available, the altitude of the target is unable to be obtained directly, either. In this research, due to the lack of the DEM data of the target zone and the flat terrain within the target zone, a flat surface is used to replace the DEM. After selecting POIs, the regular objects will be checked first. If the target point is on a known object model, then intersect the ray connecting the optical center and the video point with the model; if the target point is not on a known object model, then intersect the ray with the flat surface.

### 3.4. The Workflow of the Proposed Method

**Figure 5:**
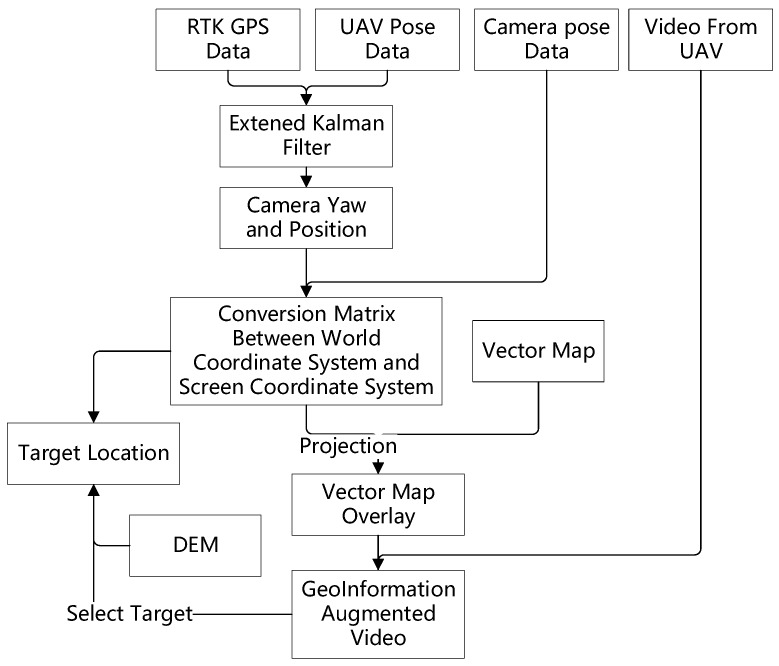
The full structure of the proposed method.

The complete workflow of the proposed method is shown in Figure 5, the RTK GPS data, the video data, the attitude data and other telemetry data of the UAV and the camera are captured by the UAV and transported to the ground in the real time when the UAV is airborne. The proposed method is composed of the following steps:

Step 1. In this step the RTK GPS data and the UAV attitude data are merged by the EKF algorithm described in Section 3.2.2, the output is the filtered UAV position sequence:

$$P_{body}=\left[\begin{array}{c} lat_{body}=\left[lat_{1},lat_{2},lat_{3}\ldots \right]\\ lon_{body}=\left[lon_{1},lon_{2},lon_{3}\ldots \right]\\ alt_{body}=\left[alt_{1},alt_{2},alt_{3}\ldots \right] \end{array}\right]$$

and UAV attitude sequence:

$$\;A_{body}=\left[\begin{array}{c} roll_{body}=\left[roll_{1}^{body},\;roll_{2}^{body},roll_{3}^{body}\ldots \right]\\ pitch_{body}=\left[pitch_{1}^{body},pitch_{2}^{body},pitch_{3}^{body}\ldots \right]\\ yaw_{body}=\left[yaw_{1}^{body},yaw_{2}^{body},yaw_{3}^{body}\ldots \right] \end{array}\right]\;$$

Since the camera is kept the same course direction as the UAV body, the yaw of the UAV body $yaw_{body}$ can be used to replace the yaw of the camera in the camera attitude sequence:

$$\;A_{camera}=\left[\begin{array}{c} roll_{camera}=\left[roll_{1}^{camera},\;roll_{2}^{camera},roll_{3}^{camera}\ldots \right]\\ pitch_{camera}=\left[pitch_{1}^{camera},pitch_{2}^{camera},pitch_{3}^{camera}\ldots \right]\\ yaw_{camera}=\left[yaw_{1}^{camera},yaw_{2}^{camera},yaw_{3}^{camera}\ldots \right] \end{array}\right]\;$$

Finally, the camera attitude sequence is:

$$\;A_{camera-new}=\left[\begin{array}{c} roll_{camera}\\ pitch_{camera}\\ yaw_{body} \end{array}\right]\;$$

Step 2. the $P_{body}$, the $A_{camera-new}$ and other necessary parameters are used to compute the conversion matrix between the world coordinate system W and the screen coordinate system S. In this process, the UAV position $P_{body}={\left[\begin{array}{ccc} lat_{body} & lon_{body} & alt_{body} \end{array}\right]}^{T}$ is converted by Equation (1). And then the conversion matrix $M_{W-S}$ is computed by Equation (2).

Step 3. In this step, the vector map from the geo-information database is projected from the geographical coordinate system to the screen coordinate system by Equation (2). The projected vector map is overlaid on the video from the UAV, to complete the augmented reality-based geo-registration of the video.

Step 4. In this step, the target is located on the screen. A point on the screen $\left(u_{s},v_{s}\right)$ can be reverse-converted to the world coordinate system using:

$$\left[\begin{array}{c} X_{s}\\ Y_{s}\\ Z_{s}\\ 1 \end{array}\right]=M_{W-S}^{T}\left[\begin{array}{c} u_{s}\\ v_{s}\\ 1 \end{array}\right]\;$$

This is a ray connecting the screen point and the optical center of the camera lenses. The coordinate of the target can be computed by intersecting this ray with the DEM of the target zone.

## 4. Dataset

A DJI M600 UAV platform (DJI, Shenzhen, China) with a RTK kit is used to test our methods in Beitun City (Xinjiang, China). The camera is a DJI Zenmuse X3 gimbal (DJI, Shenzhen, China). The gimbal is set to keep the same heading with the UAV and has a 30° downward sloping. A DJI D-RTK Kit [31] is onboard and provides the RTK GPS data. Although the D-RTK kit has two antennas, we only used the positioning result since most low-cost UAVs may be unable to carry a double-antenna RTK GPS system to determine the orientation. The RTK GPS data and other telemetry data can be obtained through the MobileSDK provided by DJI [33]. During the test, the flying height is set to 250 m above the ground level. The video stream and the telemetry data are transferred to the ground in real time and used for geo-registering and target localization. In the same time, these data are stored in local storage devices for further accuracy assessments.

**Figure 6:**
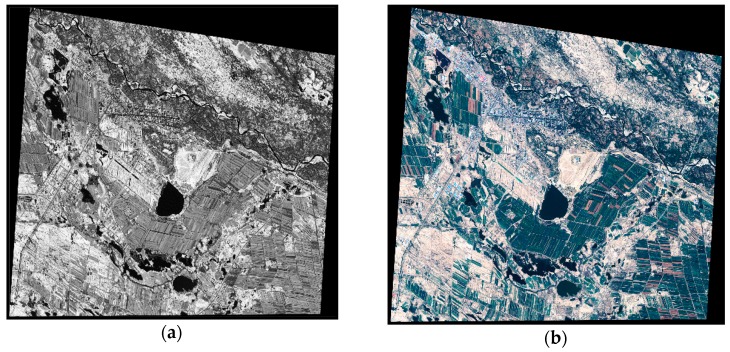
The satellite image of the test zone. (**a**) The full-color image that has 0.5 m ground resolution; (**b**) The color image that has 2 m ground resolution.

**Figure 7:**
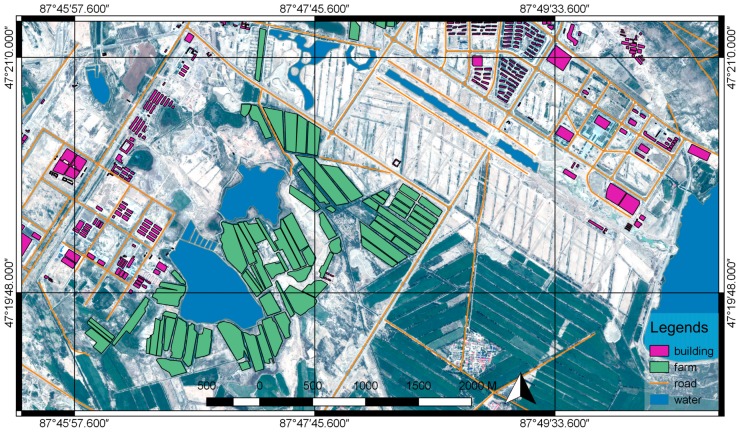
The merged 0.5 m RGB image and the vectorization result.

The vector map is obtained through the satellite images of the test zone. The satellite image dataset is taken by SPOT satellite on 14 August 2014. The dataset contains one full-color image and one RGB image. The ground resolution of the full-color image is 0.5 m, the ground resolution of the RGB image is 2 m. As shown in Figure 6, the two images are merged using ENVI to generate a 0.5 m RGB image. The vector map illustrated in Figure 7 was created from the vectorization of the 0.5 m RGB image and included roads, buildings, and waters. Because the test zone has a flat terrain, in the experiment we built a flat DSM (Digital Surface Model), this DSM is used with the vector map in the process of target localization.

## 5. Experimental Results

### 5.1. The Results of the EKF Algorithm Process

**Figure 8:**
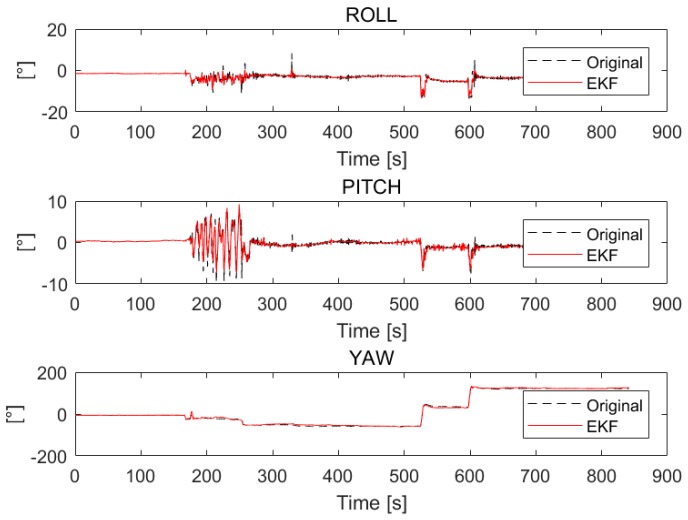
The original roll, pitch, and yaw value and the filtering result of EKF. The upper plot is the filtering result of the roll angle; the middle plot is the filtering result of the pitch angle, and the lower plot is the filtering result of the yaw angle.

The attitude data of the UAV is enhanced with the RTK GPS data using the EKF method mentioned in Section 3.2.2. The EKF algorithm requires the error parameters of the IMU sensors and the GPS receiver before the EKF processing of the data. The standard deviations of the GPS positions are 0.01 m + 1 ppm in horizontal and 0.02 m + 1 ppm in vertical according to [31]. An Allan variance analysis is performed on the IMU data from the UAV to determine the error parameters of the IMU, the results are shown in Table 1:

Because the RTK GPS is more accurate than the IMU onboard the UAV and the RTK GPS has a higher weight in the EKF process, the purpose of using RTK GPS to enhance the accuracy of the attitude data of the drone can be achieved. The enhancing results are illustrated in Figure 8.

As shown in Figure 8, the black line represents the original data of the roll, pitch and yaw angles, which is unstable. The red line represents the roll, pitch and yaw data processed by the EKF, which performs more steadily than the original data. As Table 1 indicates, the original IMU data has significant errors, that means directly using the original data will bring a considerable error to the process of the geo-registration. The following results and assessments indicate that the geo-registration and the target localization have better performance after the EKF filtering, which means the EKF filter has successfully removed the errors from the data.

### 5.2. The Result of Augmented Reality Based Geo-Registration

**Figure 9:**
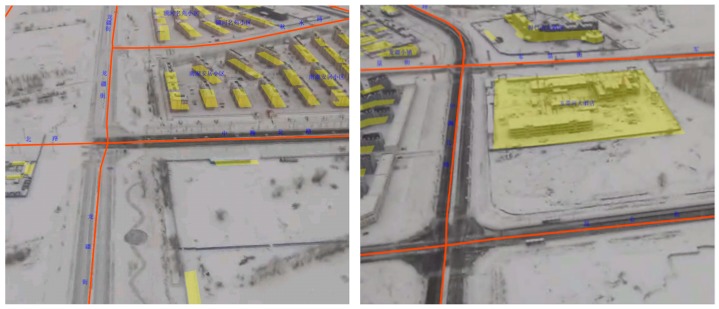
The results of the geo-registration process, the models of the roads and the buildings are matched well with the objects in the real image. (**Left**) The geo-registration process of roads and multiple buildings. (**Right**) The geo-registration process of separate large building.

Figure 9 shows the geo-registration results. The objects in the image and the vector map have no noticeable misalignment, this suggests that the proposed method can correctly calculate the conversion between the geographic coordinate system and the screen system. Because the test zone has a quite flat terrain, the flat DSM model fits the real terrain well. The orange road line of the vector map goes well with the road in the video stream, and the yellow color lumps representing the buildings are also overlaid well on the buildings in the video stream. The frame rate of the registrated video can be stabilized around 24 frames per second (fps).

## 6. Assessments and Discussion

### 6.1. Error Analysis

**Figure 10:**
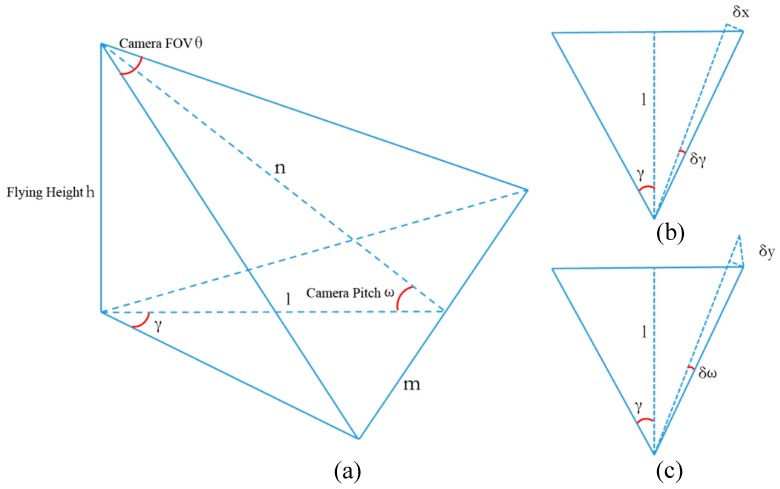
Localization error caused by roll, pitch, and yaw error. (**a**) The relationship between the horizontal FOV of the camera θ, the pitch angle of the camera ω, the height of the UAV h, and other variables. (**b**) The target localization error δx caused by the roll and yaw error δγ. (**c**) the target localization error δy caused by the pitch angle error.

Because the localization error of the UAV is less than 0.1 m, it can be ignored compared to the angular error. As shown in Figure 10, θ stands for the horizontal FOV of the camera, ω stands for the pitch angle of the camera, h is the height of the UAV. 

The camera has a mounting angle to the horizontal direction. Figure 10b presents the target localization error δx caused by the roll and yaw errors, and Figure 10c presents the target localization error δy caused by the pitch angle error.

Now let $k=\tan\frac{\theta }{2}$, according to spatial relations, we can get:

(3) $$l=\frac{h}{\tan\omega },\;n=\frac{h}{\sin\omega },\;m=\frac{\mathrm{hk}}{\sin\omega },$$

(4) $$\;\gamma =\arctan\left(\frac{m}{l}\right)=\arctan\left(\frac{k}{\cos\omega }\right),\;$$

Equation (3) is derived from ω; a differential equation is obtained:

(5) $$\;\delta \gamma =\frac{\mathrm{ksin}\omega }{k^{2}+\cos^{2}\omega }\delta \omega ,\;$$

The error of roll and yaw can be marked as δγ, the error of pitch can be marked as δω, then δ*x* and δ*y* can be calculated by:

(6) $$\;\delta x=\frac{l\delta \gamma }{\cos\gamma }=\frac{h\sqrt{\cos^{2}\omega +k^{2}}}{\sin\omega }\delta \gamma ,\;$$

(7) $$\;\delta y=\frac{\delta x}{\sin\gamma }=h\delta \omega ,\;$$

In our case, ω = 30°, θ = 60°, then k^2^ = 1/3, the expression of δ*x* can be simplified to (8):

(8)  δx=2hδγ , 

Thus, using δκ, δω, and δφ as the error of roll, pitch, and yaw, the total error caused by the attitude errors can be:

(9) $$\;\sigma =\;h\sqrt{\delta \omega^{2}+4{\left(\delta \kappa +\delta \varphi \right)}^{2}},\;$$

According to the parameters of the UAV and gimbal, δφ = 1°, δκ = δω = 1.7°, then the total error is:

(10)  σ=0.099 h, 

Thus, in the actual flight, the localization error caused by the attitude data error can reach 0.1 h. The measured values obey normal distribution in the region between 0.1 h above the actual value and 0.1 h below the real value. Filtering the data will remove the attitude values that have significant deviations from the actual values, and then narrows the distribution region to ± 0.099 h.

### 6.2. Accuracy Assessments 

**Figure 11:**
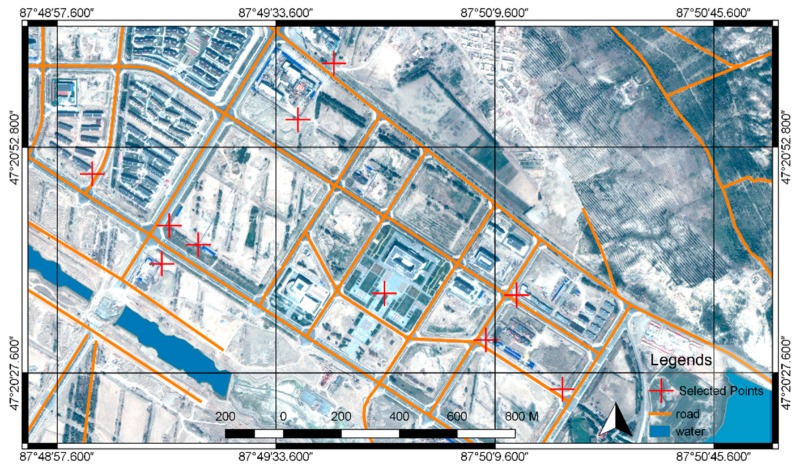
The points selected for the accuracy assessment.

**Figure 12:**
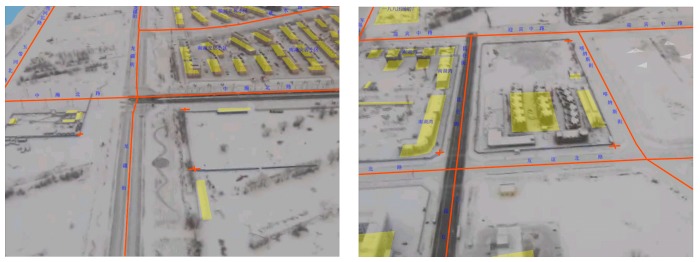
The process of target localization. The red crosses represent the target points. (**Left**) Target point 1, 2 and 3. (**Right**) Target point 8, 9 and 10.

**Figure 13:**
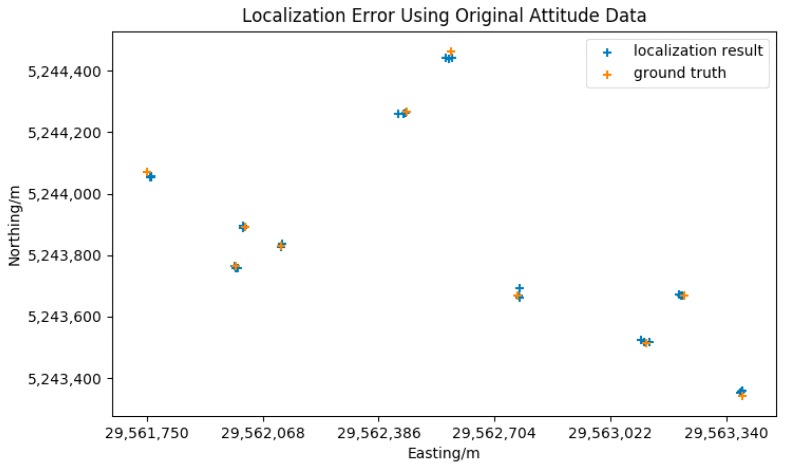
Comparison of localization results and actual geodetic coordinates.

Ten points that have noticeable features were picked up from the reference satellite image for the accuracy assessment of the target localization method. As shown in Figure 11, the points were as evenly distributed as possible in the test zone. The locations of the points from the reference satellite image were used as ground truth. During the test, the same ten points were selected manually from the video and located as shown in Figure 12. The localization results and the ground truth were projected to a flat surface using Gauss-Kruger projection. Each point was measured three times, the average error was calculated for the evaluation. The coordinates of the ground truth points and the localization results are listed in Table 2. The graphical comparison of the ground truth coordinates and the localization results is shown in Figure 13.

The comparison in Table 2 shows that the localization results have significant error compared to the ground truth. The errors are not stable and vary enormously. As the original attitude data has considerable noise, this pattern of error is reasonable. According to Equation (10), the maximum error of the localization error may reach 24.75 m, the errors using original attitude data are in the calculated range. Figure 13 indicates that the error shows no apparent direction pattern either. This also fits well with the significant noise of the attitude data.

**Figure 14:**
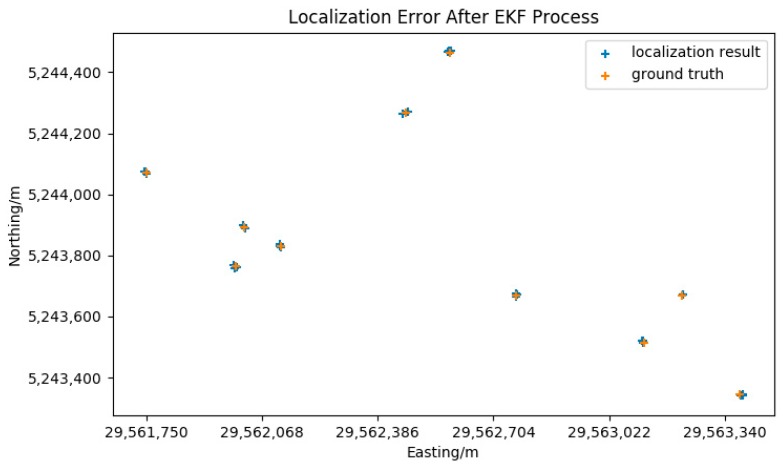
Comparison of localization results and ground truth coordinates, after EKF processing.

To reduce the error of the attitude data, the RTK GPS was used to enhance the attitude data by the EKF algorithm mentioned in Section 3.2.2. The coordinates of the ground truth points and the localization results after EKF are listed in Table 2. The graphical comparison of the ground truth coordinates and the localization results after EKF is shown in Figure 14.

The result shown in Table 2 indicates that the localization error after applying the EKF decreases significantly. The RMS (Root Mean Square) error decreases from 12.58 m to 5.21 m. The accuracy and the distribution of the localization results are both improved. The P value of two-tailed *t*-test between the two sets of localization errors is 0.0042, which indicates that the difference is statistically significant. The significant accuracy improvement after using the EKF enhances yaw data for the camera attitude, proving that the yaw value relying on the magnetic compass is a major source of the geo-registration error. However there still exist errors that not decreased by the EKF process, these errors may come from flowing sources:

- The mounting position error. The mounting position error consists of two parts: the offset between the GPS antenna and the center of the drone, and the offset between the center of the drone and the camera optical center. The flight controller of the M600 drone has a built-in compensation mechanism for the offset between the GPS antenna and the center of the drone. Due to the existing of the gimbal, the camera has position and angle offset from the center of the drone. The measurement of the camera position may be not accurate. Furthermore, to isolate the high-frequency vibration from the motors, there are several vibration absorbers between the gimbal and the drone body. The soft connection between the camera and the drone will lead the mounting angle between the UAV body and the camera to be varied during the flight, which will subsequently make the yaw angle of the drone differ from the yaw angle of the camera.
- The video lag. During the test, the telemetry data and the video data are transferred through different data links. The video link has a delay in the transfer process, this may cause the telemetry data aligning to a wrong video frame and bring errors to the geo-registration and target localization process. The onboard video link has a latency of 50 ms [34] and may be more according to environmental conditions. The base latency of 50 ms was compensated in the experiments and a test zone that has low radio inference was selected in order to minimize the effects of video lag. During the flight, the attitude and position data may not arrive at the same time as the video frame, this difference is compensated by applying a linear extrapolation between contiguous attitude and position data frames.
- The screen selecting error. Affected by the screen resolution and the human operation, the position selected on the screen may not the exact point of the target, these errors may be several meters in the real world.
- The rough terrain model. During the flight test, the terrain of the test zone is represented by a flat surface. As the actual terrain varies and the orientation of the UAV changes. The value and direction of the error may change irregularity. When there exists DEM data of the target zone, using actual DEM of the target zone can increase the localization accuracy.

### 6.3. The Use of DJI M600 Platform

This article intends to demonstrate a method for getting satisfying augmented reality geo-registration result and target localization result on low-cost UAV platforms. However, the price of DJI M600 platform is slightly higher than most electronic multi-rotor drones. The reason for choosing the DJI M600 platform is as follows:

- Although the DJI M600 is slightly more expensive among multi-rotor UAV platforms, the difficulty and cost of using the M600 are still much lower than that of oil-powered helicopter UAVs, which are capable of carrying navigation-level IMU sensors. A single person can operate an M600 in extreme situations that is nearly impossible for oil-powered helicopter UAVs.
- The D-RTK kit onboard the M600 is an off-the-shelf product provided by DJI, which is highly integrated with the flight controller of the M600. This experiment platform can save plenty of time and work from building a test environment from scratch. Although the M600 is not very cheap compared to cheap quadcopters, the attitude sensors of M600 and the onboard camera gimbal have no essential difference with the cheap drones. Thus the conclusions of this research are also applicable to cheap quadcopters. The proposed method in this article used none of the extra abilities that only an expensive platform like the M600 can have. There is no theoretical barrier to apply the proposed method to a real low-cost UAV platform.

## 7. Conclusions

This paper presents an augmented reality geo-registration method for geo-registering video streams from low-cost UAVs and ground targets localization. In the proposed method, a conversion model between the world coordinate system and the screen system was used to complete the augmented reality-based geo-registration of the video. The RTK GPS data was used to enhance the body attitude data by the EKF algorithm, the camera yaw data was replaced by the enhanced body yaw data in the geo-registration process to improve the accuracy of geo-registration. A target localization method based on the geo-registration model was proposed to complete the target localization process on the video. The performance of the proposed method was demonstrated by a case study in Beitun City, Xinjiang Province, China. The results showed that the proposed method performed well in the test environment. In the augmented video, the geometries and the marks were placed in the correct place with the corresponding objects in the video, and the attitude data of the drone was enhanced efficiently by the EKF algorithm. The target localization results were improved with the enhanced attitude data of the drone and the camera.

The limitation of this study is that in the real world the terrain is not a simple flat surface. Using a flat surface ignores the slight changes of the ground, which brings errors to the localization results. The DEM of the working zone in the real world needs to be used to acquire a better localization accuracy. The EKF fusion between the RTK GPS data and the UAV attitude data improves the accuracy of the attitude data significantly. However, there still exists systematical error between the actual yaw value and the fusion result, which will have to be calibrated strictly. This error may vary with the working zones and latitudes, which is not easy to be solved in practical applications. The vector data and the ground truth points are not quite accurate, as such the localization results are relative values. Since the system doesn’t have a double antenna RTK GPS system, the augmentation of the attitude data using the RTK GPS data requires the UAV to be moving to ensure a satisfactory result. When the UAV is hovering, the fusion performance may decrease. In such circumstances, manual interaction can be introduced to improve the geo-registration accuracy.

## Figures and Tables

**Table 1:** The error parameters of the IMU determined by the Allan variance analysis.

Direction	Angle Random Walks $\left(\times 10^{-4}\;{}^{\circ}/\sqrt{\mathrm{hour}}\right)$	Velocity Random Walks $\left(\times 10^{-3}\;m/s/\sqrt{\mathrm{hour}}\right)$	$\mathrm{Gyroscope}\;\mathrm{Dynamic}\;\mathrm{Biases}\;(\times 10^{-5}\;{}^{\circ}/s)$	$\mathrm{Accelerometer}\;\mathrm{Dynamic}\;\mathrm{Biases}\;(\times 10^{-4}\;\mathrm{mg})$	Gyroscope Correlation Times	Accelerometer Correlation Times
X	5.481	3.6	6.35	7.610	50	30
Y	5.907	9.3	12.78	3.055	20	60
Z	5.717	9.3	1.12	4.012	60	40

**Table 2:** Comparison of localization errors using original attitude data and EKF filtered attitude data.

Result Set					Errors/m						RMS Error/m
Using Original Attitude Data	4.94	5.28	4.15	13.67	12.33	10.89	12.19	18.48	22.11	8.74	12.58
EKF Filtered Attitude Data	3.86	5.33	3.56	5.50	4.62	5.55	5.63	5.57	7.32	4.03	5.21
